# Correlation between Myocardial Velocity Measured using Tissue Doppler Imaging in the Left Ventricular Lead-Implanted Segment and Response to Cardiac Resynchronization Therapy

**DOI:** 10.6061/clinics/2019/e1077

**Published:** 2019-09-26

**Authors:** Dong-Mei Yang, Fei Yu, Kang-Yu Chen, Hao Su, Qi Wang, Zhi-Quan Liu, Kai Hu, Jian Xu, Ji Yan

**Affiliations:** Division of Cardiology, First Affiliated Hospital of the University of Science and Technology of China, Hefei, Anhui, China

**Keywords:** Cardiac Resynchronization Therapy, Heart Failure, Echocardiography, Tissue Doppler Imaging

## Abstract

**OBJECTIVES:**

This study investigated whether tissue Doppler imaging parameters, especially the peak systolic velocity of the left ventricular lead-implanted segment (Ss), affect cardiac resynchronization therapy response.

**METHODS:**

In this case-control study, 110 enrolled patients were divided into cases (responder group, n=65) and controls (nonresponder group, n=45) based on whether their left ventricular end-systolic volume was reduced by ≥15% at 6 months after surgery. Preoperative clinical and echocardiographic data were collected. Multivariate logistic regression models were used to analyze the factors affecting the response to cardiac resynchronization therapy, and receiver operating characteristic curves were plotted to evaluate their diagnostic values.

**RESULTS:**

The proportion of patients with left bundle branch block in the case group was higher than that in the control group. The control group showed a higher left atrial volume index, E/A ratio and E/Em ratio but lower Ss than that of the case group. A multivariate regression analysis showed that left bundle branch block, Ss, and an E/Em ratio>14 were independent risk factors affecting the response to cardiac resynchronization therapy. Ss=4.1 cm/s was the best diagnostic threshold according to the receiver operating characteristic curve.

**CONCLUSIONS:**

Ss is an important factor affecting the response to cardiac resynchronization therapy. Patients with heart failure associated with Ss<4.1 cm/s have a higher risk of nonresponse.

## INTRODUCTION

With the recent accumulation of clinical evidence-based data and the popularization of cardiovascular intervention techniques, the number of patients with heart failure (HF) receiving implanted cardiac resynchronization devices in China has increased annually. In addition, the implantation technique has gradually become available in primary-care medical centers. However, despite strictly following the inclusion criteria in the guidelines, approximately 30% of recipients do not respond to cardiac resynchronization therapy (CRT) 1. If the efficacy of CRT is assessed only using the degree of left ventricular (LV) remodeling, then the nonresponse rate is even higher 2. Therefore, quickly and accurately identifying potential nonresponders to CRT before surgery is of great clinical significance. Given the advantages of noninvasive, easy-to-use techniques that are sensitive to local myocardial motion and less affected by preloading, tissue Doppler imaging (TDI) is widely used to diagnose and treat cardiovascular diseases 3. Many clinical studies have shown that in normal and HF populations, TDI-evaluated myocardial tissue velocity (peak systolic [Sm], early diastolic [Em], late diastolic [Am], and others) has a strong predictive value for major adverse cardiovascular events 4-9. However, whether the aforementioned TDI parameters (especially the myocardial tissue velocity of the segment containing the LV lead implant) affect response to CRT remains unknown. Thus, the present study conducted a retrospective case analysis to investigate the relevant factors affecting response to CRT and determine the parameters for ultrasound screening in clinical practice.

## METHODS

The Institutional Review Board of the First Affiliated Hospital of the University of Science and Technology of China approved this retrospective observational study.

Between August 2015 and December 2016, patients with HF undergoing CRT at our hospital were included in this study based on the following inclusion and exclusion criteria. The inclusion criteria were 1 patients with New York Heart Association (NYHA) Heart Function Class II to IV after routine treatment; 2 sinus rhythm; 3 patients with left bundle branch block (LBBB) and a QRS duration ≥130 ms or without LBBB but with a QRS duration ≥150 ms; and 4 an LV ejection fraction (LVEF) ≤35%. Patients were excluded when they had 1 an upgraded or replaced pacemaker; 2 quadripolar lead or multipoint pacing; 3 uncompleted heart TDI examinations or poor-quality ultrasound images; 4 no cardiac ultrasound data 6 months after surgery; or 5 right bundle branch block (RBBB).

Baseline data including age, gender, height, weight, HF etiology, NYHA classification, blood biochemistry profile (creatinine and NT-proBNP levels), and therapeutic agents were collected from the electronic medical records system. Heart rate, QRS duration, and QRS morphology were evaluated using 12-lead ECG before surgery. LV lead position was determined by reviewing the surgical notes.

**Figure 1:**
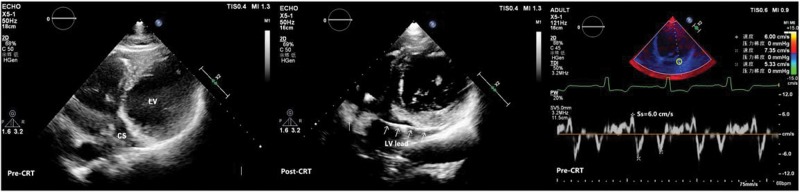
Example of myocardial velocity curve via tissue Doppler imaging. The sample was obtained from the left ventricular lead-implanted segment (yellow circle); the Y-axis represents myocardial velocity (cm/s), and the X-axis represents time; Ss is the positive peak on the curve.

The preoperative and 6-month postoperative echocardiographic indices were evaluated using a Philips iE33 ultrasound system with an X5-1 transducer (1-5 MHz). Ultrasound examination was performed following the American Society of Echocardiography (ASE) guidelines 10. Left atrial and LV volumes were measured using Simpson's biplane method and indexed to body surface area. The left atrial volume index (LAVI), LV end-diastolic volume index (LVEDVI), and LV end-systolic volume index (LVESVI) were documented. Preoperatively, the mitral flow spectrum was acquired using pulse Doppler imaging, and the peak early diastolic velocity (E) and peak late diastolic velocity (A) were recorded. TDI was used to obtain the myocardial velocity curves of the lateral and septal mitral annulus and 12 LV segments (six basal and six middle segments). Sm, Em, Am, mitral annular velocities, and the peak systolic velocity of the LV lead-implanted segment (Ss) were recorded (Figure 1). Intra- and interobserver reproducibility for Ss were evaluated with regard to 20 randomly selected patients and expressed as an intraclass correlation coefficient (ICC). The intraobserver and interobserver ICCs were 0.98 and 0.97, respectively.

A reduction ≥15% in LVESV 6 months after surgery was defined as a response to CRT. Patients with or without a response to CRT were assigned to the case and control groups, respectively.

SPSS 22.0 was used for data analysis. Continuous variables with normal distributions were expressed as means±standard deviations. Continuous variables with nonnormal distributions were expressed as medians and interquartile ranges (serum creatinine and NT-proBNP levels). Independent-samples t-tests (variables with normal distributions) or Mann-Whitney U tests (serum creatinine and NT-proBNP) were used to compare between-group differences. Paired Student's t-tests were used to compare data within groups. Categorical variables were expressed as frequencies and percentages. The chi-square test or Mann-Whitney U test (NYHA classification) was used to compare between-group differences. Factors associated with significant differences in the univariate analysis (*p*<0.05) were included in the multivariate logistic regression model. The backward stepwise entry method (LR method) was used to screen relevant factors affecting response to CRT. The receiver operating characteristic (ROC) curve was used to evaluate the diagnostic value of Ss in predicting response to CRT. The maximum value of the Youden index was used as the optimal diagnostic threshold to calculate sensitivity and specificity. A two-tailed *p*-value of <0.05 was considered as significant.

## RESULTS

**Figure 2:**
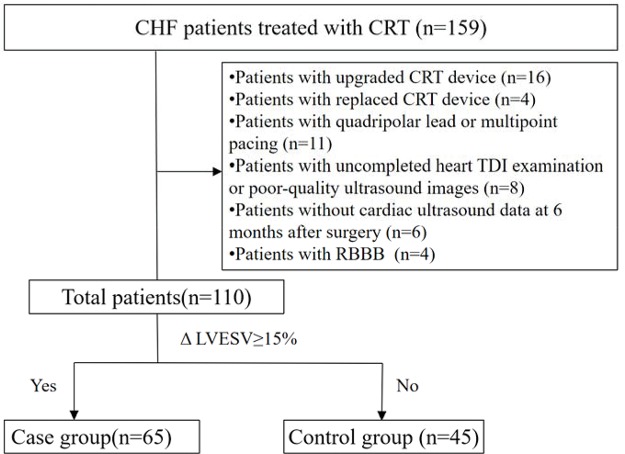
Study flow diagram.

Figure 2 shows the flow diagram of this study. Of the 159 patients with HF who received CRT at our hospital between August 2015 and December 2016, 110 were included in this study (mean age: 61.7±10.2 years; women: 30%; CRT-D: 72%) and were assigned to either the case group (65 patients, 59%) or the control group (45 patients).

The baseline data for the case and control groups are shown in Table 1. No significant differences were found with regard to gender, age, causes of HF, NYHA classification, heart rate, QRS duration, serum creatinine level, NT-proBNP level, echocardiographic indices (LVEDVI, LVESVI, LVEF, E, A, Sm, Em and Am), medication regimen or LV lead position between the two groups (*p*>0.05 for all comparisons). The proportion of patients with LBBB in the case group was higher than that in the control group (72% *vs*. 42%, χ^2^=10.028, *p*=0.002). In addition, the control group showed a higher LAVI, E/A ratio, and E/Em ratio but lower Ss than the case group (*p*<0.05 for all comparisons).

**Figure 3:**
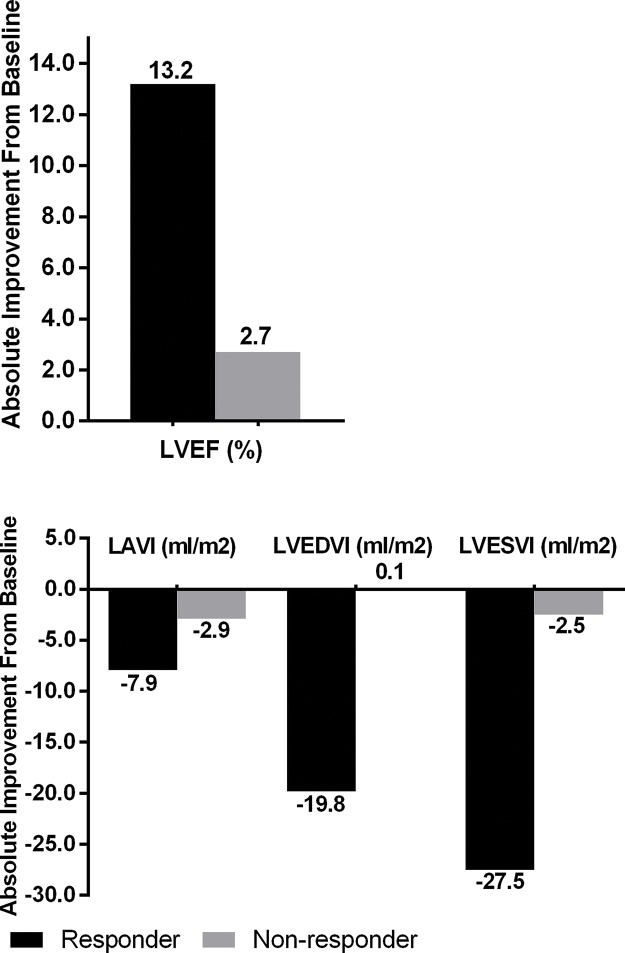
Changes in echocardiographic parameters in responder and nonresponder groups.

Table 2 and Figure 3 show the changes in echocardiographic indices 6 months after surgery in both groups. Six months after the surgery, the LAVI, LVEDVI, LVESVI, and LVEF were significantly improved in the case group (*p*<0.001 for all comparisons), whereas only the LVEF was improved in the control group (*p*<0.001).

Table 3 shows the logistic regression analysis results. Of the 15 factors included in the univariate analysis, LBBB, the LAVI, E/A ratio, Ss, and an E/Em ratio >14 were significantly associated with CRT response (*p*<0.05 for all comparisons). An additional multivariate regression analysis showed that LBBB (OR: 4.193; 95% CIs: 1.697-10.361), Ss (OR: 1.548; 95% CIs: 1.078-2.222) and an E/Em ratio >14 (OR: 0.326; 95% CIs: 0.131-0.814) were independent risk factors affecting CRT response. Because Ss might be affected by age, age was included in the multivariate regression model. However, the inclusion of age in the model did not lead to a significant change (<10%) in the OR of Ss; therefore, the data were not adjusted for age 11.

**Figure 4:**
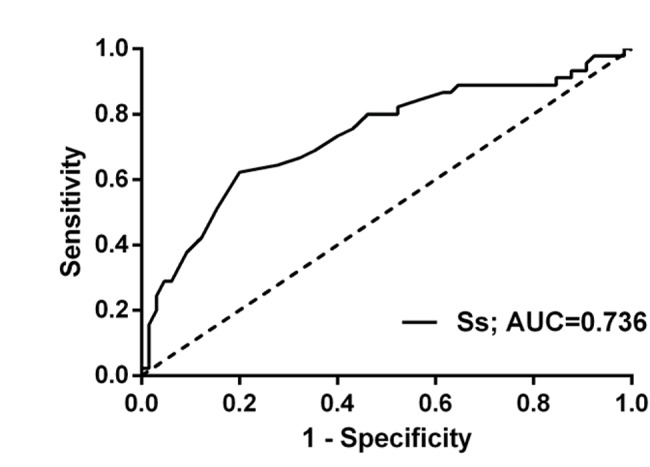
Receiver operating characteristic (ROC) curve of Ss to predict CRT response.

Figure 4 shows the ROC curve for Ss predicting CRT response. The area under the curve (AUC) was 0.736 (95% CIs: 0.638-0.835), and Ss=4.1 cm/s was the best diagnostic threshold for the ROC curve. The corresponding sensitivity, specificity, positive predictive value (PPV), and negative predictive value (NPV) were 80%, 64%, 77%, and 69% respectively.

## DISCUSSION

This study revealed that 1 preoperative LBBB, Ss, and an elevated LV filling pressure (E/Em >14) were independent risk factors affecting CRT response and that 2 patients with HF with Ss <4.1 cm/s have a higher risk of nonresponse to CRT.

Should TDI be used to assess the maximum difference in time to peak systolic velocity or peak velocity? TDI has been used to screen patients suitable for CRT. In most studies, LV mechanical asynchrony was evaluated based on the peak time difference measured via TDI. Previous single-center studies with small sample sizes have also found that certain ultrasound synchronic indicators (i.e., Ts-[lateral-septal], 12Ts-SD, PVD, and others) have predictive value for CRT efficacy 12-14. However, even if examiners with uniform training measure and analyze these indicators, the reproducibility of these measurements remains unsatisfactory. It is also difficult for the sensitivity and specificity of the indicators to meet the requirements of extensive clinical application. Therefore, a prospective, multicenter study (PROSPECT) did not recommend using the above ultrasound synchronization indices as screening criteria for CRT 2.

This result is not surprising because the greatest advantage of TDI is its sensitivity to local myocardial motion 9, whereas it is relatively insensitive to whole heart movement. Many evidence-based clinical studies have shown that in either normal or HF populations, TDI-evaluated myocardial tissue velocity (Sm, Em, Am, and so on) has a strong predictive value for major adverse cardiovascular events (e.g., cardiovascular death, nonfatal myocardial infarction and HF exacerbation) 4-9. It remains unclear whether the aforementioned indicators can be used to assess CRT efficacy in populations with moderate-to-severe HF. This study revealed that Ss is an independent risk factor affecting CRT response and that patients with Ss <4.1 cm/s might have a higher risk of nonresponse to CRT. Moreover, the traditionally used indicators (e.g., Sm, Em, and Am) have no significant predictive value for CRT response. The LV lead position is the key to determining CRT efficacy. A reduced Ss reflects a decrease in the myocardial viability in this region, suggesting the presence of local ischemia, fibrosis, or scars 15,16 that affect the benefits of CRT. This finding cannot be achieved via the assessment of myocardial motion at the mitral annulus, which is distant from the lead. This study also suggests that surgeons should avoid implanting LV leads in regions with Ss <4.1 cm/s. If avoiding these areas is impossible because of variant target veins, then quadripolar LV leads, LV multipoint pacing, or LV intracardiac pacing should be used to “bypass” the low-viability region of the myocardium to help increase CRT response rates.

In this study, preoperative LBBB and E/Em >14 were independent risk factors affecting CRT response. Both QRS duration and QRS morphology are important factors affecting CRT efficacy 17. However, this study found that only LBBB morphology affects the benefit of CRT. The reasons for this result might be related to the patients with HF included in this study because they had wide QRS waves (baseline QRSd=162 ms) according to the guideline criteria; therefore, an additional increase in QRS duration cannot significantly increase CRT response rate. Biagio Sassone et al. 18 also reported that a baseline QRSd >178 ms is associated with a lower CRT response rate. A 10-year prospective, multicenter study by Gasparini et al. 19 included 3,319 patients undergoing CRT and found that a baseline QRSd ≧200 ms was associated with an increased risk of all-cause and cardiac mortality.

The E/Em ratio is a robust indicator of LV filling pressure in ultrasound assessment. An E/Em ratio >14 is highly specific, indicating increased LV filling pressure 3, which signifies a worsening hemodynamic state, increased heart transplant rate, and higher mortality among patients with HF; hence, it is a strong predictor of poor prognosis 20,21. In this study, increased LV filling pressure was also a risk factor for nonresponse to CRT. This finding is consistent with Ciampi Q et al. 22, which further validates the results of our previous study 23.

### Limitations

This study has the following limitations. 1 It was designed as a case-control study and included consecutive patients treated in our hospital; however, selection bias is difficult to avoid, and our conclusions must be further verified by prospective studies. 2 Patients implanted with quadripolar leads or MPP were excluded because the variability in pacing sites made it difficult to measure Ss. 3 Speckle-tracking technology can detect more abundant myocardial motion patterns (including longitudinal, radial, and toroidal motion) and might help increase CRT response rates 24. However, speckle-tracking technology requires specific equipment, highly skilled examiners, and professional software for offline analysis; thus, it is difficult to extensively use this technology in primary-care medical centers. This study aimed to determine a rapid preoperative screening indicator for medical centers at all tiers; therefore, speckle-tracking technology was not applied. 4 The reduction of myocardial velocity assessed via TDI can only indicate decreases in myocardial viability in the corresponding region. This study did not use cardiac magnetic resonance or cardiac PET to further investigate the reasons underlying reduced myocardial viability.

## CONCLUSIONS

In summary, the present study showed that preoperative LBBB, Ss, and elevated LV filling pressure are independent risk factors that affect CRT response. Furthermore, patients with HF with Ss <4.1 cm/s have a higher risk of not responding to CRT. Therefore, surgeons should avoid implanting the LV lead in segments with significant Ss reduction to increase CRT response rate.

## AUTHOR CONTRIBUTIONS

Yang DM, Yu F and Yan J conceived and designed the study. Yang DM and Yu F drafted the manuscript. Yu F, Wang Q, Liu ZQ and Hu K were responsible for the data acquisition. Yang DM, Chen KY and Su H were responsible for the data analysis and interpretation. Xu J and Yan J provided financial support. Yan J provided administrative support. Su H, Xu J and Yan J critically revised the manuscript for important intellectual content.

## Figures and Tables

**Table 1:** Baseline characteristics of patients.

	Responders (n=65)	Nonresponders (n=45)	*p-*value
**Clinical parameters**			
Age, years	60.6±10.6	63.1±9.4	0.207
Female, n (%)	19 (29)	14 (31)	0.832
Ischemic etiology, n (%)	16 (25)	13 (29)	0.617
NYHA class, n (%)			0.581
II	12 (18)	9 (20)	
III	35 (54)	26 (58)	
IV	18 (28)	10 (22)	
Heart rate (beats/min)	74.5±12.6	70.6±10.2	0.096
LBBB, n (%)	47 (72)	19 (42)	0.002
QRS duration, ms	163.3±20.3	160.7±18.3	0.486
Serum creatinine, μmol/l	89.0 (33.0)	83.0 (37.0)	0.481
NT-proBNP, pg/mL	1,825.0 (1,673)	2,274.0 (2,369.0)	0.091
**Echocardiography**			
LAVI, ml/m2	42.4±17.0	52.8±19.7	0.004
LVEDVI, ml/m2	126.8±50.9	125.8±44.0	0.914
LVESVI, ml/m2	93.8±39.3	91.9±34.3	0.784
LVEF, %	26.2±4.5	27.2±5.6	0.290
**Pulse Doppler**			
E, cm/s	71.4±21.5	80.3±31.0	0.068
A, cm/s	65.4±24.6	58.5±21.7	0.133
E/A ratio	1.3±0.6	1.6±0.9	0.031
**Tissue Doppler**			
Ss, cm/s	5.1±1.5	4.2±1.4	0.001
Sm, cm/s	4.9±1.3	4.5±1.1	0.091
Em, cm/s	5.8±1.9	5.2±1.5	0.068
Am, cm/s	6.4±2.1	6.0±1.8	0.287
E/Em ratio	12.9±4.7	16.5±7.1	0.005
**Medication**			
ACE-I or ARB, n (%)	58 (89)	41 (91)	1.000
β-Blocker, n (%)	57 (88)	36 (80)	0.272
Spironolactone, n (%)	60 (92)	42 (93)	1.000
**LV pacing site**			0.611
Anterolateral, n (%)	7 (11)	4 (9)	
Lateral, n (%)	42 (65)	33 (73)	
Posterolateral, n (%)	16 (25)	8 (18)	

Values are n (%), mean±SD, or median (interquartile range).

ACE-I *=* angiotensin-converting enzyme inhibitor; ARB *=* angiotensin receptor blockers; LAVI *=* left atrial volume index; LBBB *=* left bundle branch block; LVEDVI *=* left ventricular end diastolic volume index; LVEF *=* left ventricular ejection fraction; LVESVI *=* left ventricular end systolic volume index; NYHA *=* New York Heart Association; RBBB *=* right bundle branch block.

**Table 2:** Echocardiographic characteristics of patients at baseline and follow-up.

	Responders (n=65)		Nonresponders (n=45)	
	Baseline	Follow up	Baseline	Follow up
LAVI, ml/m2	42.4±17.0	34.5±15.1*	52.8±19.7	49.9±15.4
LVEDVI, ml/m2	126.8±50.9	107.0±43.3*	125.8±44.0	125.7±48.0
LVESVI, ml/m2	93.8±39.3	66.3±32.0*	91.9±34.3	89.4±35.7
LVEF, %	26.2±4.5	39.4±9.0*	27.2±5.6	29.9±6.2*

* p<0.001, follow up vs. baseline; see Table 1 for abbreviations.

**Table 3:** Univariate and multivariate logistic regression analyses: Estimates of the correlations between baseline clinical and echocardiographic characteristics and CRT response.

Parameter	Univariate		Multivariate	
	OR (95% CIs)	*p-*value	OR (95% CIs)	*p-*value
Age, years	0.975 (0.939-1.014)	0.207		
Female	0.915 (0.400-2.091)	0.832		
Ischemic etiology	0.804 (0.341-1.893)	0.617		
NYHA class IV	1.340 (0.551-3.259)	0.518		
LBBB	3.573 (1.600-7.977)	0.002	4.193 (1.697-10.361)	0.002
QRS duration, ms	1.007 (0.987-1.028)	0.484		
NT-proBNP, pg/mL	1.000 (1.000-1.000)	0.197		
LAVI, ml/m2	0.969 (0.948-0.991)	0.006		
E, cm/s	0.985 (0.970-1.000)	0.056		
E/A ratio	0.547 (0.321-0.933)	0.027		
Ss, cm/s	1.682 (1.190-2.376)	0.003	1.548 (1.078-2.222)	0.018
Sm, cm/s	1.335 (0.952-1.873)	0.094		
Em, cm/s	1.247 (0.980-1.587)	0.073		
E/Em ratio>14	0.366 (0.165-0.814)	0.014	0.326 (0.131-0.814)	0.016
LV pacing site	1.177 (0.590-2.351)	0.643		

OR = odds ratio; CIs = confidence intervals; see Table 1 for additional abbreviations.
